# Impact of Mg Doping on Structural, Morphological and Thermoelectric Properties of SnO_2_ Nanoparticles: A Combined Experimental-Theoretical Investigation

**DOI:** 10.3390/molecules30204135

**Published:** 2025-10-20

**Authors:** Muhammad Isram, Matteo Barduzzi, Valeria Demontis, Daniele Goldoni, Pino D’Amico, Luigi Rovati, Alberto Vomiero, Alice Ruini, Francesco Rossella

**Affiliations:** 1 Dipartimento di Scienze Fisiche, Informatiche e Matematiche, University of Modena and Reggio Emilia, Via Campi 213/A, 41125 Modena, Italy; 2 Department of Physics, University of Cagliari, 09042 Monserrato, Italy; 3 Istituto Nanoscienze CNR-NANO-S3, Via Campi 213/A, 41125 Modena, Italy; 4 Division of Materials Science, Department of Engineering Sciences and Mathematics, Luleå University of Technology, SE-971 87 Luleå, Sweden

**Keywords:** nanotechnology, thermoelectricity, density functional theory, Raman

## Abstract

Recent advances in nanotechnology, including the development of nanoparticles, thin films, and superlattices, have revitalized research in thermoelectricity by enabling independent control of thermal and electrical transport, overcoming longstanding efficiency limitations and expanding opportunities for sustainable energy generation and miniaturized device applications. Tin dioxide (SnO_2_) has recently attracted increasing attention as a thermoelectric material owing to its properties, such as high-temperature chemical and structural stability, non-toxicity, and the abundance of constituent elements. Current research efforts have been directed toward enhancing its thermoelectric performance through strategies such as elemental doping, nanostructuring, strain engineering, and the development of composite systems. In this study, we investigate the effects of Mg substitutional doping on the thermoelectric characteristics of SnO_2_. We synthesize undoped and Mg-doped SnO_2_ nanoparticles (0.05%, 0.10%, and 0.15%) using a straightforward hydrothermal technique. The investigation of the undoped and doped materials revealed that SnO_2_ possesses a tetragonal rutile-type structure, as determined through structural and morphological examination. The crystalline size of all of the samples decreases as the Mg doping concentration is increased. Hall measurement and Seebeck coefficient measurements have been employed for assessing the thermoelectric characteristics. As the Mg content increased, both the Seebeck coefficient and electrical conductivity value increased from −20 μV/K to −91 μV/K and 29.8 S/cm to 112.6 S/cm, confirming the presence of semiconductor behavior. The 0.15% Mg-doped sample demonstrates the highest power factor when evaluated at a temperature of 150 K, yielding a value of 9.4 $\times \;10^{-5}$ WK^−2^m^−1^.

## 1. Introduction

In the past few decades, the direct conversion of heat into electricity through thermoelectric materials has been extensively investigated as a promising route toward sustainable energy generation [1,2,3,4]. Thermoelectric devices have found application in power generation, micro-cooling systems for electronics, and waste heat recovery, owing to their solid-state nature and scalability [5,6]. Nevertheless, the widespread adoption of thermoelectric technologies remained hindered by the limited efficiency of existing materials. Thermoelectricity, once regarded as constrained by fundamental material limitations, experienced a remarkable revival over the past decade, driven by advances in the field of nanotechnologies and by the growing need for devices to power small-scale devices, such as wearable, implantable, biomedical, and chip integrated devices and harness waste heat at the nanoscale. Nanostructuring strategies, including the synthesis of nanoparticles [7], thin films [8,9], and nanowires [10,11,12,13], has enabled unprecedented control over phonon scattering and carrier transport, thereby decoupling thermal and electrical properties. These breakthroughs have allowed for the design of materials with significantly reduced thermal conductivity without severely compromising electrical performance, opening new perspectives for the optimization of thermoelectric materials across various temperature ranges.

The evaluation of a material’s suitability for thermoelectric applications is governed by the dimensionless figure of merit ZT, defined as $ZT=S^{2}\sigma T/\kappa$, where *S* is the Seebeck coefficient of the material, σ is the electrical conductivity, *T* is the absolute temperature, and κ is thermal conductivity, which can be expressed as the sum of two contributions: $\kappa_{e}$ for the electron contribution and $\kappa_{p}h$ for the phonon contribution. An ideal thermoelectric material should therefore possess high *S* and σ, and low thermal conductivity.

Classic inorganic materials such as Bi_2_Te_3_, PbTe, SiGe, and SnSe have long been the standard due to their relatively high thermoelectric performance, especially near room temperature [1]. However, recent research has expanded to include half-Heusler alloys, multicomponent oxides, organic–inorganic composites, carbon nanomaterials, and conducting polymers [14,15,16]. Moreover, there has been significant interest in semiconductor oxide materials due to their exceptional characteristics, including high-temperature chemical and structural stability, non-toxicity (making them environmentally friendly), and the abundance of constituent elements. Some examples include layered cobalt oxides, perovskite-structured compounds, and transparent conducting oxides, among other possibilities. Several p-type and n-type oxide semiconductors, including Ca_3_Co_4_O_9_, NaxCoO_2_, LaCoO_3_, SrTiO_3_, CaMnO_3_, ZnO, TiO_2_, and SnO_2_, have been investigated in previous studies [17,18,19,20,21,22]. Nevertheless, the ZT values exhibited by these materials remain insufficient for practical use [22], which typically requires ZT > 1.

Tin dioxide is a wide-bandgap semiconductor ($E_{g}$ = 3.6–3.8 eV) and exhibits a tetragonal rutile crystalline structure. SnO_2_, along with impurity-doped SnO_2_, is primarily utilized in the manufacturing of transparent conducting coatings, solid-state gas sensors, and heterogeneous catalysts, due to its remarkable electrical, optical, and structural properties [23]. In recent literature, multiple authors have documented the favorable thermoelectric properties exhibited by routinely sintered ceramic materials based on SnO_2_, particularly in the context of n-type oxide materials [24,25]. Doping with different elements has emerged as a potential technique for enhancing the thermoelectric performance of SnO_2_ [26]. Doping SnO_2_ nanoparticles with magnesium (Mg) is a promising strategy for enhancing their thermoelectric performance. Mg incorporation can significantly modify their electrical and thermal transport properties by affecting carrier concentration, mobility, and phonon scattering, possibly leading to improved thermoelectric characteristics [27]. Specifically, substitutional Mg^2+^ doping at Sn^4+^ sites in SnO_2_ is believed to create oxygen vacancy Vo^2+^ [28,29]. The presence of these vacancies plays a crucial role in facilitating the movement of high-energy charge carriers, hence significantly influencing the thermoelectric material’s overall thermoelectric performance.

In this work, we systematically investigate the effects of Mg doping on the structural, morphological, and thermoelectric properties of SnO_2_ nanoparticles synthesized via a hydrothermal approach [30,31]. This technique is a straightforward method to fabricate pure and Mg-doped SnO_2_ nanoparticles, which provides numerous advantages, such as relatively low growth temperatures, the possibility to exactly manipulate the stoichiometry, and the attainment of high levels of porosity and purity. Particular attention is devoted to correlating the induced microstructural modifications with the measured electrical conductivity, Seebeck coefficient, and overall power factor enhancement, providing insights for the rational design of high-performance oxide thermoelectric materials. Furthermore, ab initio simulations are carried out to validate the transport properties of this system, such as the electrical conductivity, Seebeck coefficient, and the power factor, in order to have a more general understanding of the electron transport in the bulk SnO_2_ crystal structure.

## 2. Results and Discussion

### 2.1. Structural Characterization

**Figure 1:**
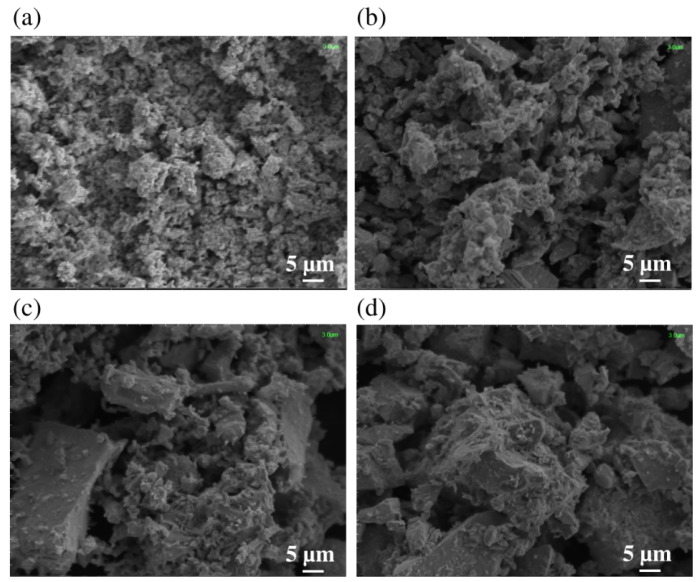
SEM images of undoped SnO_2_ and Mg-doped nanoparticles: (**a**) undoped SnO_2_, (**b**) 0.05% Mg-doped, (**c**) 0.10% Mg-doped, and (**d**) 0.15% Mg-doped.

Figure 1 displays the scanning electron microscopy (SEM) microstructures and surface morphology of undoped SnO_2_ nanoparticles and Mg-doped SnO_2_ samples containing varying concentrations of Mg. As shown in the Figure 1, the morphology strongly depends on the Mg concentration. The undoped SnO_2_ nanoparticles showed a relatively smooth surface, and a sudden increase in the roughness of the microstructures is observed as the Mg concentration is increased. The presence of nano-sized grains can still be observed on the surface of the Mg-doped SnO_2_ nanoparticles.

**Figure 2:**
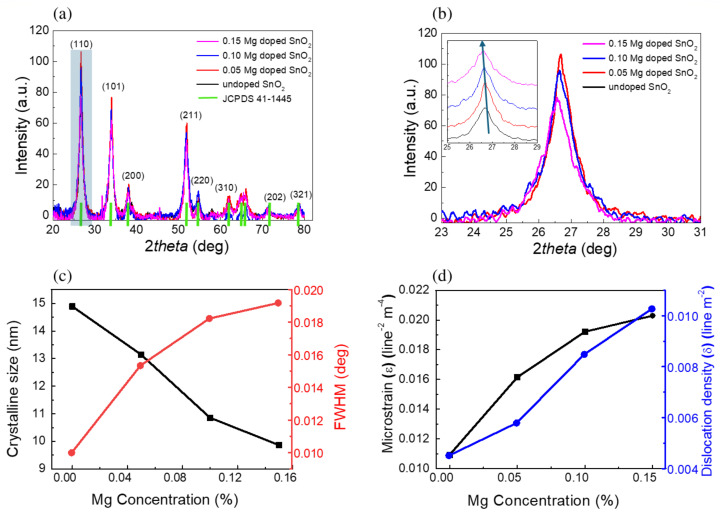
(**a**) XRD spectra of undoped SnO_2_ nanoparticles and SnO_2_ nanoparticles doped with varying Mg concentrations; (**b**) (110) peak (not-normalized, corresponding to the highlighted peak in (**a**)). Inset: vertically shifted curves highlighting the peak shift; (**c**) Variation of the crystalline size and FWHM as a function of the doping concentration; (**d**) microstrain and dislocation density for undoped and Mg-doped SnO_2_ nanostructures.

The phase crystallinity and structural analysis of undoped and Mg-doped SnO_2_ nanoparticles were determined by using the non-destructive X ray diffraction technique, as shown in Figure 2. Figure 2a displays the raw data and Figure 2b displays the curves with a vertical offset to make the features more visible. The X-ray investigation revealed that both the undoped and doped Mg:SnO_2_ nanoparticles exhibited similar XRD patterns with characteristic peaks of (110), (101), (200), (211), (220), (221), (310), (202), and (321), indicating a tetragonal rutile phase structure, in agreement with the standard JCPDS data (file no: 41-1445). There are no other phases observed, such as metallic Mg, SnO_2_ orthorhombic phase, SnO, or MgO-based phases.

As is evident in the magnification of the (110) peak spectral region (Figure 2b, curved not normalized), we observed that increasing M-doping promotes a decrease in the (110) peak intensity, associated with the replacement of Sn^4+^ ions with Mg^2+^. The inset reports the same curves with a vertical shift, together with the curve for the undoped sample, showing the occurrence of an angular shift among the peaks.

The observed variations in the XRD peak intensity and position can be attributed to the substitution of Sn^4+^ by Mg^2+^ ions in the crystal lattice. In general, the intensity of diffraction peaks reflects the degree of crystallinity, where sharper and more intense peaks indicate improved long-range order. The incorporation of Mg^2+^, having a different ionic radius and charge state compared to Sn^4+^, may introduce local strain and disorder within the lattice, which can reduce crystallinity and lead to broadening or a decrease in peak intensity [32,33]. On the other hand, peak position shifts are directly related to changes in the lattice parameters. Since Mg^2+^ (0.72 Å) has a smaller ionic radius compared to Sn^4+^ (0.69 Å for six-fold coordination, but with higher charge density), its substitution alters the bond lengths and induces lattice distortion. This results in systematic peak shifts in the diffraction patterns, reflecting unit cell contraction or expansion depending on the substitution mechanism and local defect chemistry [34,35].

As the Mg content increases, a small shift towards lower angles (in the inset, the (110) peak position is displayed) is observed, which was previously attributed to the stress induced by the incorporation of Mg^2+^ ions at Sn^4+^ sites. In fact, the Sn^4+^ ion has a slightly larger radius (0.071 nm) than the Mg^2+^ ion (0.067 nm) [36,37], although they are sufficiently similar enough to guarantee an easy substitution.

Figure 2c shows the average crystallite size of undoped and Mg-doped SnO nanoparticles, determined using the Scherrer formula [38] given by

(1) $$D=\frac{K\lambda }{\beta \cos\theta }$$

where *D* is crystallite size, *K* represents shape factor and has a value 0.89, λ is the beam wavelength, β indicates full width at half maximum (FWHM) and θ is the Bragg angle. The results showed that SnO_2_ has an average crystalline size of 15 nm. The results clearly show that Mg substitution in the Sn sites inhibits grain growth and the crystallite size decreases with doping. Figure 2d displays the variation in the microstrain and dislocation density for different Mg concentrations. These parameters were determined through the following equations [39]:

(2) $$\epsilon =\frac{\beta }{4}\cos\theta$$

(3) $$\delta =\frac{1}{D^{2}}$$

The results reported in Table 1 show that the inclusion of Mg induces both a reduction in the crystallite size and increasing stress in the lattice, likely due to the the small difference in the ionic radii of the two elements. Table 1 in fact shows that both the microstrain and dislocation density are enhanced in the Mg-doped samples.

### 2.2. Raman Analysis

**Figure 3:**
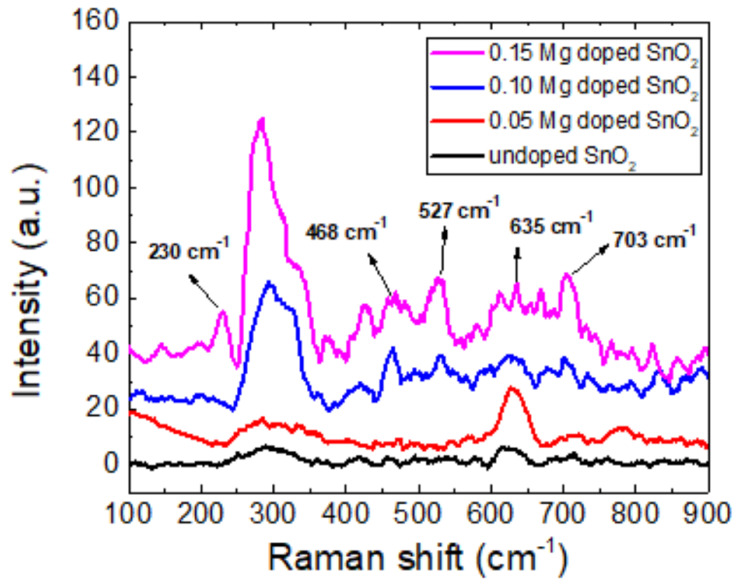
Raman spectra for undoped and Mg-doped SnO_2_ samples with doping concentrations of 0.05%, 0.1%, and 0.15% Mg (measurements performed at room temperature).

Raman spectroscopy is a powerful non-destructive technique that is utilized for analyzing a wide variety of material quality characteristics, such as the degree of disorder, crystal structure, and flaws in doped semiconductor oxides. The Raman effect, or inelastic light scattering, is the foundation of Raman spectroscopy. According to a number of studies, the SnO_2_ lattice will frequently create the following major vibration modes: $\Gamma =A_{1g}+A_{2g}+B_{1g}+B_{2g}+E_{g}+A_{2u}+2B_{1u}+3E_{u}$. In this case, the infrared active modes are $A_{2u}$ and $E_{u}$, and the inactive modes are $A_{2g}$ and $B_{1u}$. The Raman active modes are $A_{1g}$, $B_{1g}$, $B_{2g}$, and $E_{g}$. One characteristic of the $A_{1g}$ mode is the simultaneous stretching of each Sn-O bond along a direction that preserves the symmetry of the crystal. On the other hand, the Sn-O bond is stretched asymmetrically in the $B_{2g}$ mode, which disrupts the symmetry of the crystal. Figure 3 shows the Raman spectra collected for the pure SnO_2_ sample as well as the doped ones. While in the pure SnO_2_ nanoparticles’ spectra, active Raman modes at 230 and 468 cm^−1^ were predicted [40], only the mode at 468 cm^−1^ is weakly present in the experimental spectrum. However, the incorporation of magnesium promotes the emergence of both modes, particularly the one at 230 cm^−1^, although it is slightly shifted toward higher wavenumbers. Due to the presence of a slightly mismatched Mg^2+^ ion in the SnO lattice, the intensity of these modes also rises when magnesium is introduced into the material. The $A_{1g}$ mode and $B_{2g}$ mode are other detectable Raman active modes of pure SnO_2_ at 635, 527, and 703 cm^−1^, respectively. The tetragonal phase of SnO_2_ can be used to index the recorded Raman modes of $A_{1g}$ and $B_{2g}$ [41]. By increasing the Mg doping concentration in SnO_2_, it can be observed that the peaks with intensities of magnitudes of $A_{1g}$ and $B_{2g}$ changed in an unpredictable manner.According to findings from earlier investigations, the variation in intensity may be connected to the materials’ synthesized crystallite size [42]. As a result, both the Raman spectra and XRD patterns supported the appearance of the tetragonal rutile phase of SnO_2_.

### 2.3. Thermoelectric Properties

**Figure 4:**
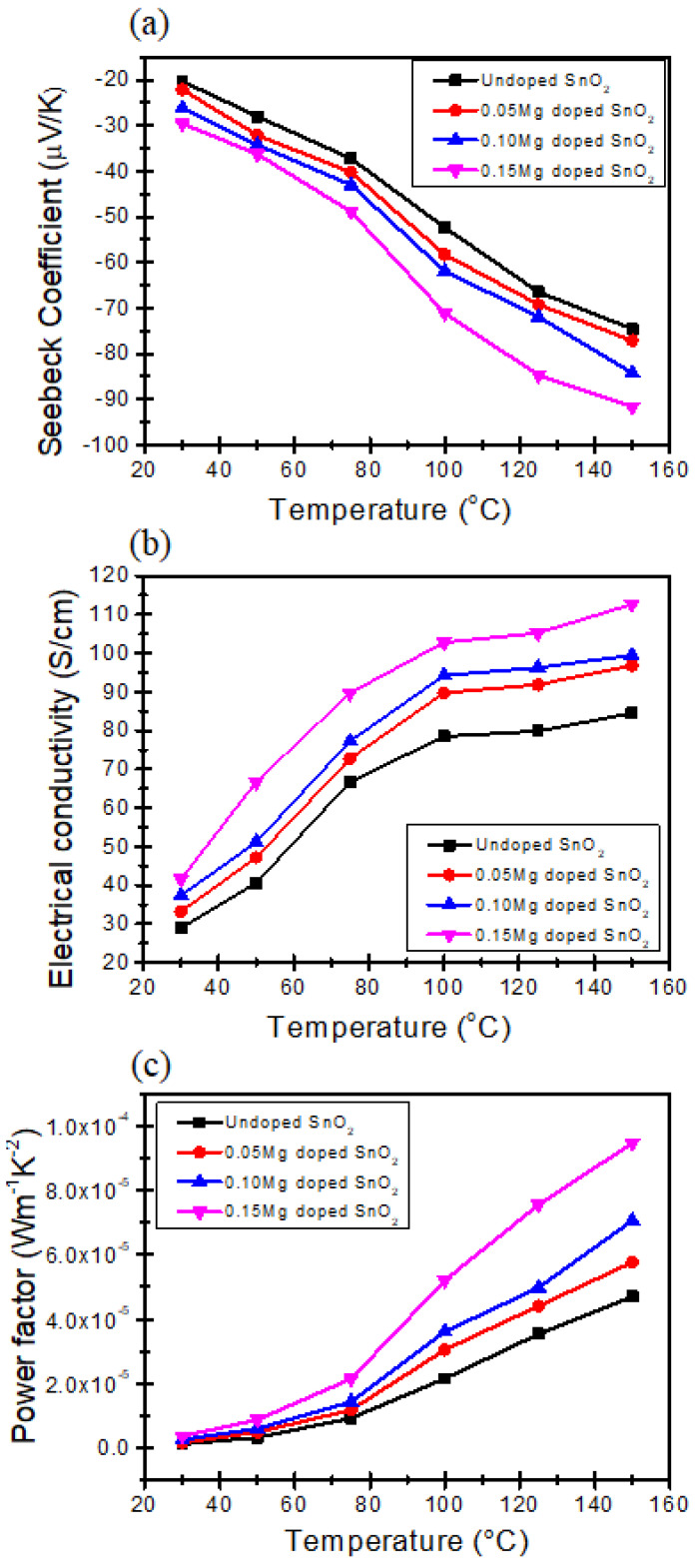
Temperature dependence of (**a**) Seebeck coefficient, (**b**) electrical conductivity, (**c**) power factor of pure SnO_2_ and Mg-doped SnO_2_ nanoparticles.

In order to assess the thermoelectric (TE) characteristics of the synthesized samples, we initially transformed them into pellets with a cylinder shape, univocally characterized by their diameter and height. The typical pellet dimensions were 2.50 cm diameter and 1.50 cm height. The Seebeck coefficient value was subsequently assessed in relation to the absolute temperature range of 30 °C to 150 °C, as depicted in Figure 4a. The samples exhibit a negative Seebeck coefficient, indicating the presence of n-type electronic conductivity.

As the concentration of Mg was raised, a decrease in the Seebeck coefficient value is observed, from −20 μV/K to −91 μV/K. Moreover, the Seebeck coefficient also decreases with rising temperature. In Table 1, it is observed that the crystalline size of SnO_2_-doped Mg decreases as the SnO_2_ content increases. Consequently, Figure 4a demonstrates that the Seebeck coefficient of SnO_2_-doped Mg exhibits a corresponding increase. The Seebeck coefficient of SnO_2_ experiences a notable enhancement when the Mg concentration increases, and this enhancement becomes more pronounced as the Mg level in the samples increases. The addition of Mg to the samples leads to an increase in electrical conductivity. Additionally, there is an inverse relationship between the Seebeck coefficient and the charge carrier concentration. This suggests that the decrease in the Seebeck coefficient for the doped samples can be attributed to a higher carrier concentration [15]. In a broad context, it has been observed that there is an inverse relationship between the increase in electrical conductivity and the decrease in the Seebeck coefficient [43]. The findings of our study suggest that the conduction mechanism observed in the SnO_2_-doped Mg samples cannot be entirely explained by a traditional model rooted in band theory. Instead, it appears to be influenced by a significant electron–electron correlation effect, as supported by previous research [44]. The electrical conductivity of the synthesized samples was measured using Hall measurements (Figure 4b). The electrical conductivity of stoichiometric SnO_2_ should be quite low due to the large bandgap. Nevertheless, n-type behavior is often observed due to native defects, especially oxygen vacancies [45]. The increase in the Mg concentration in SnO_2_ resulted in the enhancement of the electrical conductivity, with values increasing from 29.8 S/cm to 112.6 S/cm, which also confirms the semiconducting behavior. The introduction of Mg^2+^ in place of Sn^4+^ has a similar effect as the introduction of an impurity donor or dopants that donate two electrons to the crystal: the increased electron concentration offsets the electric charge equilibrium, resulting in the enhancement of the crystal electrical conductivity. In other words, the substitution of Mg^2+^ for Sn^4+^ in SnO_2_ introduces a charge imbalance in the lattice that acts analogously to intentional impurity doping. This imbalance perturbs the charge neutrality condition, forcing the system to compensate by generating charge carriers (electrons or holes), which directly impacts the material’s electrical conductivity. In many semiconducting oxides, such aliovalent substitution is a well-established strategy for controlling electron concentration and tailoring transport properties. In this regard, ref. [46] demonstrated in high-quality epitaxial SnO_2_ thin films that donor doping (e.g., Sb substituting Sn) increases the free electron density and enhances conductivity by introducing extra electrons into the conduction band. Similarly, ref. [47] investigated Mg doping in SnO_2_ and confirmed that Mg^2+^ substitution at Sn^4+^ sites alters the electronic structure and carrier balance: Mg incorporation compensates donor defects such as oxygen vacancies, leading initially to a reduced electron concentration and eventually to a conduction-type transformation (from n-type to p-type) depending on the Mg content. Thus, the introduction of Mg^2+^ in place of Sn^4+^ can indeed be understood within the same framework as donor/acceptor doping changing the carrier concentration to offset charge imbalance and thereby modifying the electrical conductivity. The carrier mobility was observed to rise as the measurement temperature was raised from 303 K to 423 K, resulting in an increase in the electrical conductivity of all the samples. Figure 4c) displays the experimental results of the temperature dependency of the power factor (PF) = $S^{2}\sigma$ for the undoped and Mg-doped SnO_2_ nanoparticles, with different Mg concentrations of 0.05, 0.10, and 0.15. The power factor of the Mg-doped SnO_2_ nanoparticles exhibits an increase up to a temperature of 150 K. The power factors exhibited by the Mg-doped SnO_2_ nanoparticles are significantly greater when compared to the power factors observed in the pure SnO_2_ sample. The thermoelectric power factor of the Mg-doped SnO_2_ sample reaches its maximum value of 9.4 × 10^−5^ WK^−2^m^−1^ at a temperature of 423 K.

## 3. Experimental Section

### 3.1. Materials

The chemicals used in this study, namely, tin chloride dihydrate (SnCl_2_ · 2H_2_O), magnesium dichloride (MgCl_2_), and sodium hydroxide (NaOH), were acquired from Sigma Aldrich (St. Louis, MO, USA) with a purity of 98.9%. The experiment utilized commercially acquired acetone, ethanol, deionized water, and other compounds.

### 3.2. Synthesis of Mg Doped SnO_2_ Nanoparticles

**Figure 5:**
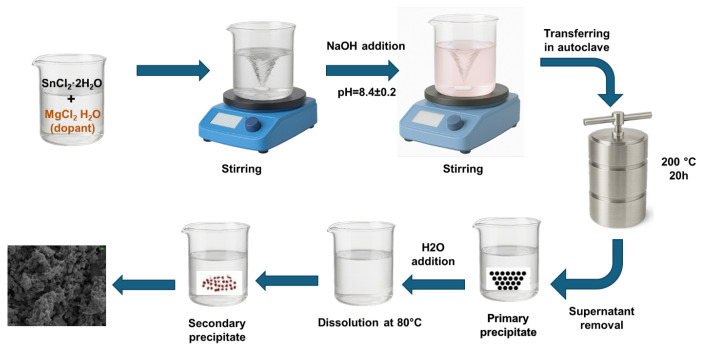
Schematics of the hydrothermal process used for synthesizing undoped and Mg-doped SnO_2_ nanoparticles.

A solution was prepared by dissolving one mole of SnCl_2_·2H_2_O in 50 mL of deionized water at a temperature of 60 °C for a duration of 1 h, while maintaining the mixture under continuous stirring. The doping solution used in this study was MgCl_2_ dissolved in a prepared solution containing Sn_1−x_Mg_x_O_2_ nanoparticles. The values of x used in the experiment were 0%, 0.05%, 0.10%, and 0.15%. During stirring, a solution of NaOH was incrementally added drop by drop in order to raise the pH level to a range of 8.4 ± 0.2. The prepared solution was then transferred into an autoclave, which was then placed for 20 h inside an oven at a temperature of 200 °C, in order to facilitate additional chemical reactions. A similar process was used for the synthesis of all samples. After the completion of the chemical reaction, the resultant solution was cooled to ambient conditions, and the precipitates were separated thorough a rinsing process involving ethanol, acetone, and deionized water. Figure 5 displays the schematic diagram illustrating the growth process.

### 3.3. Characterization

The X-ray diffraction patterns were acquired using a Bruker D8 Advance diffractometer equipped with Cu Kα radiation (λ = 1.5406 Å), with a 2θ scanning range of 10–90°, step size of 0.02°, and scan speed 1°/min. The vibrational modes of the samples were investigated using a Raman spectrometer (Dongwoo Opteron, Gwangju-si, Republic of Korea) coupled with a laser source operating at a wavelength of 632 nm with a power of the laser aimed at the sample of 1 mW, spectral resolution of 1 cm^−1^, acquisition time of 10 s (number of accumulations: 3), objective lens of 50×, and CCD detector. The morphology of the samples was characterized using the scanning electron microscope (SEM) (Emcrafts, Hanam-si, Republic of Korea). The Seebeck coefficient, which is influenced by temperature, was determined using a self-designed Seebeck system for thermoelectric study, where the sample is placed between two copper blocks acting as hot and cold contacts. A resistive micro-heater generates a controlled temperature gradient (ΔT), which is monitored using two calibrated K-type thermocouples attached directly to the sample ends. The corresponding thermovoltage (ΔV) is measured with a nanovoltmeter (Keithley 2182A, Cleveland, OH, USA) via low-resistance gold probes. The Seebeck coefficient is calculated as S = −ΔV/ΔT. The overall measurement uncertainty is within 3–5%, ensuring reliable and reproducible results. Additionally, the measurement of electrical conductivity was conducted using the Ecopia 3000 Hall apparatus (Anyang-si, Republic of Korea).

## 4. Theoretical Investigation

**Figure 6:**
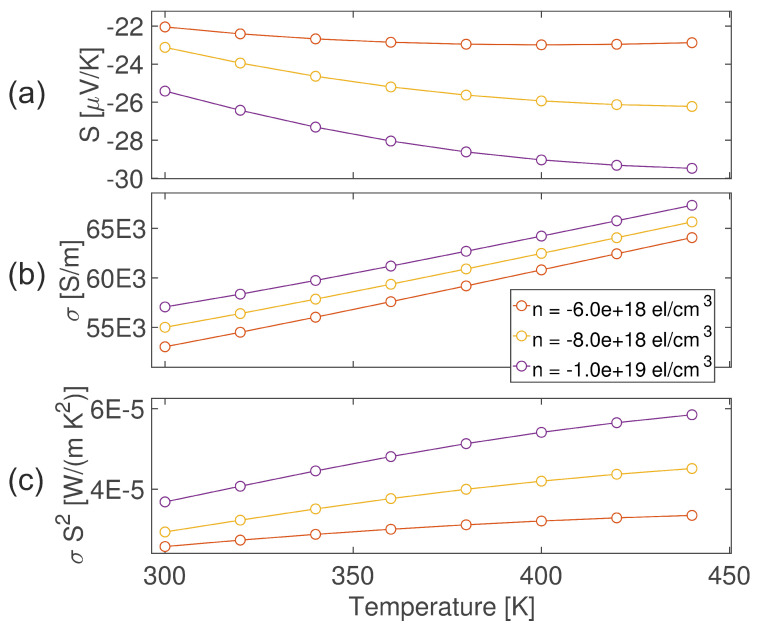
(**a**) Seebeck coefficient, (**b**) electrical conductivity, and (**c**) power factor obtained from the solution of the Wigner Transport Equation on 25 × 25 × 25 k-mesh.

To support the interpretation of the experimental results from a theoretical point of view, ab initio calculations based on Density Functional Theory (DFT) have been performed in order to obtain the electronic transport properties of the SnO_2_ crystal. Density Functional Perturbation Theory (DFPT) has been employed to calculate the phonon eigenmodes and the electron–phonon coupling tensor, and the latter has been used to calculate the electron–phonon scattering matrix, considered the main component of electron scattering at room temperature. The semiclassical Wigner Transport Equation (WTE) has been chosen for the description of transport properties, motivated by the “anomalous” behavior of the Seebeck coefficient for different carrier concentrations (see Figure 6a). The WTE, in contrast to the Boltzmann Transport Equation (BTE), can be more accurate for strongly anharmonic systems, narrow-gap semiconductors, or strong dipole coupling like in this case. It is described by the following equation:

(4) $$\frac{\partial F(r,k,t)}{\partial t}+i\left[\epsilon \left(k\right)+d\left(k\right)\;\cdot \;E,F\left(r,k,t\right)\right]+\frac{1}{2}\left\{V\left(k\right),\;\cdot \;\nabla_{r}F\left(r,k,t\right)\right\}-eE\;\cdot \;\nabla_{k}F\left(r,k,t\right)=\frac{\partial F(r,k,t)}{\partial t}{|}_{coll},$$

where F(r,k,t) is the electron quasiprobability distribution, which describes the quantum system; the operators [,] and {,} are the commutator and anticommutator respectively; ϵ(k) is the matrix of energies; and d(k) is the electric dipole. It is important to mention that the right hand side of Equation (4) includes the scattering matrix, which includes the diagonal terms (the same of the Boltzmann Transport Equation) and the out-off diagonal terms, which are considered to be the coupling of multiple bands. The BTE is the limit of the WTE when the out-off diagonal terms of the scattering matrix are orders of magnitude lower with respect the diagonal terms.

To solve the DFT and DFPT problems, a plane wave basis set and optimized norm-conserving Vanderbilt Pseudopotentials (ONCVPP) [48] have been employed along with the GGA-PBE approximation [49] for the exchange–correlation functional, as implemented in the Quantum Espresso suite of codes [50]. The self-consistent solution was obtained with an accuracy of 1 × 10^−14^ Ry for the total energy, after the full relaxation of the SnO_2_ primitive cell, with a force convergence threshold of 1 × 10^−3^ Ry/Bohr and a pressure convergence threshold of 0.5 kBar. For the Brillouin zone sampling, a 16 × 16 × 16 Monkhorst–Pack grid has been used, with 110 Ry for the energy cutoff of the wave function. For the phonon calculation, the same energy convergence threshold was adopted, using a q-mesh of 4 × 4 × 4. The maximally localized Wannier functions (MLWFs) were used as implemented in Wannier90 code [51] to interpolate the electronic band structure to solve the so-called Wannier–Wigner Transport Equation (WWTE), projecting Bloch’s state on the sp and sp3 orbitals of Sn and on the sp orbital of O, with a convergence up to $10^{-6}{\overset{˚}{A}}^{2}$. The solutions of the WWTE, as implemented in the Phoebe code [52], were obtained on a 25 × 25 × 25 k-mesh, with different values of temperature in a range from 250 K to 400 K, and carrier concentrations in a range of $10^{18}$ to $10^{20}$ electrons/cm^3^ (producing fake Mg doping, and therefore different chemical potential (The values of the carrier concentration were selected in order to cause the same behavior of the calculated transport coefficient with respect to the experimental ones)), using the adaptive Gaussian smearing method for the Dirac delta approximation (see [52] for the details). The overall transport calculations were performed within the rigid band approximation, since the percentage of Mg doping was too low to modify the electronic DOS, as was also reported in previous DFT calculations [53].

**Figure 7:**
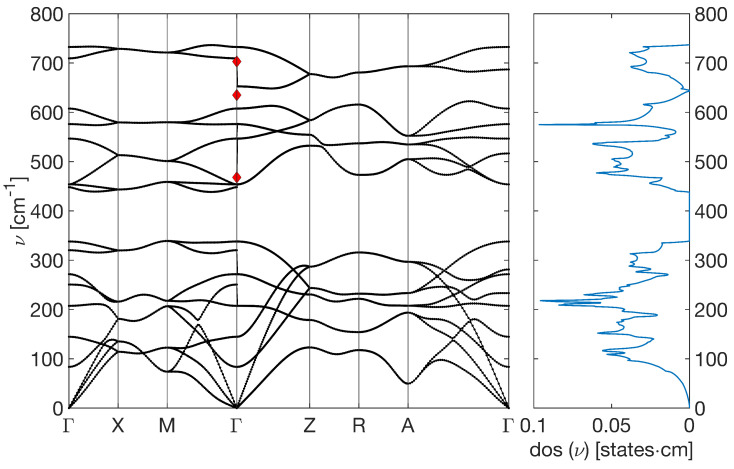
Phonon bands and DOS of SnO_2_ pristine sample, calculated from DFPT. The red diamonds are the values obtained experimentally.

Figure 7 reports the calculated phonon density of states and the band structure, for which the Raman peaks are obtained at 734 cm^−1^, 609 cm^−1^, and 456 cm^−1^ for the $B_{2g},A_{1g}$ and $E_{G}$ peak, respectively, which are in good agreement with the experimental values and also with other similar calculations [54].

The Seebeck coefficient calculated from the WWTE is reported in Figure 6a. Comparing these results with the experimental ones reported in Figure 4a, it is possible to see that for carrier concentrations below 1 × 10^19^ electrons/cm^3^, the temperature dependence of the Seebeck coefficient can be reproduced. Furthermore, the carrier concentration increase contributes to raise (in absolute value) the Seebeck coefficient, as reported in the experiment results; this behavior is in conflict with Mott’s formula, for which the Seebeck coefficient of a degenerate semiconductor can be approximated as $S\propto \frac{T}{n^{2/3}}$, where *n* is the free carrier density and *T* is the temperature. The failure of the latter formula could be attributed to the fact that, within the Wigner transport theory, the electron mobility of the system tends to decrease for high doping, in contrast to the BTE, for which at a higher value of carrier concentration, the mobility of the system increases. Moreover, the dipole coupling could cause the inversion of Mott’s equation, increasing the Seebeck coefficient with increasing free carrier density. The underestimation of the calculated Seebeck coefficients with respect to the experimental one for higher temperatures could be attributed to the overestimation of the electrical conductivity due to the presence of other scattering mechanisms not considered in our framework for the computational complexity, like boundary and impurity scattering, which are expected to be relevant in nanoparticles at higher temperatures, as also described in Section 2.1.

Moreover, when the carrier concentration reaches 2 × 10^19^ el/cm^3^ (not reported in Figure 4),there is an inversion in the temperature dependence of the Seebeck coefficient (it decreases in absolute value when the temperature rises). For this reason, the range of carrier concentrations that reproduces the experimental results is the one below 2 × 10^19^ el/cm^3^.

The electrical conductivity calculated from the WWTE is reported in Figure 6b. The values are, in general, overestimated with respect to the experimental ones by an order of magnitude, as expected since the only scattering mechanism considered is electron–phonon coupling; however, the general temperature and carrier concentration dependencies are reproduced well, as expected from the general semiconductor theory.

Lastly, the power factor is shown in Figure 6c. As expected, the calculated values are overestimated with respect to the experimental ones, due to the already discussed overestimation of σ. Also, in this case, the carrier concentration that reproduces the experimental behavior is the one below 2 × 10^19^ el/cm^3^.

## 5. Conclusions

Mg-doped SnO_2_ nanoparticles were synthesized by hydrothermal techniques with a crystalline size range of 14.8–9.8 nm, and thermoelectric studies were carried out, employing a multi-technique approach. The XRD measurements indicate a pure tetragonal rutile structure, also confirmed by the Raman spectra, with the crystalline size decreasing with increasing Mg concentration. The SEM imaging correlates the increase in the Mg doping concentration to the sample roughness, with pure SnO_2_ nanoparticles having a smooth, crystal-like surface.The electrical transport measurements reveal increased electrical conductivity with increasing Mg doping, tentatively ascribable to the enhanced electron density associated with Mg ion incorporation. The Seebeck coefficient’s absolute value was also found to increase with the Mg content, and similarly, the power factor, with the compound Sn_0.85_Mg_0.15_O_2_ displaying a power factor of 9.4 × $10^{-5}$ Wm^−1^K^−2^ at 150 K. The ab initio calculations (Density Functional Theory, Density Functional Perturbation Theory, and Wannier Functions to address Wigner Transport Equation for bulk SnO_2_) show a good correlation with the experimental data, with slight overestimation of the electrical conductivity (Seebeck underestimation), providing the transport coefficients for carrier concentrations between 6 × 10^18^ and 2 × 10^19^ cm^−3^.

## Figures and Tables

**Table 1:** The calculated structural parameters of undoped and Mg-doped SnO_2_ with different doping concentrations: 0 to 0.15%.

Mg Doping	FWHM (Degree)	Crystalline Size D (nm)	Inter-Planar Spacing (d)	Microstrain (ϵ)	Dislocation Density (δ) $\times \;10^{-3}$
Pure	0.0099	14.8	3.455	0.0109	4.512
0.05	0.0153	13.2	3.334	0.0161	5.791
0.10	0.0182	10.8	3.337	0.0192	8.485
0.15	0.0191	9.8	3.349	0.0203	10.271

## Data Availability

The original contributions presented in this study are included in the article. Further inquiries can be directed to the corresponding author(s).
